# A62 EXAMINING THE ACTIVATION OF XENOBIOTIC RECEPTORS PXR AND AHR IN COLONOIDS USING MICROBIAL METABOLITES AND CHEMICAL LIGANDS

**DOI:** 10.1093/jcag/gwad061.062

**Published:** 2024-02-14

**Authors:** Eva I Shenoda, Simon A Hirota

**Affiliations:** SNYDER INSTITUTE FOR CHRONIC DISEASES, UNIVERSITY OF CALGARY, CALGARY, AB, CANADA; ALBERTA CHILDREN’S HOSPITAL RESEARCH INSTITUTE, UNIVERSITY OF CALGARY, CALGARY, AB, CANADA; DEPARTMENT OF PHYSIOLOGY AND PHARMACOLOGY, UNIVERSITY OF CALGARY, CALGARY, AB, CANADA; SNYDER INSTITUTE FOR CHRONIC DISEASES, UNIVERSITY OF CALGARY, CALGARY, AB, CANADA; ALBERTA CHILDREN’S HOSPITAL RESEARCH INSTITUTE, UNIVERSITY OF CALGARY, CALGARY, AB, CANADA; DEPARTMENT OF PHYSIOLOGY AND PHARMACOLOGY, UNIVERSITY OF CALGARY, CALGARY, AB, CANADA; DEPARTMENT OF MICROBIOLOGY, IMMUNOLOGY & INFECTIOUS DISEASES, UNIVERSITY OF CALGARY, CALGARY, AB, CANADA

## Abstract

**Background:**

The Aryl hydrocarbon receptor (AhR) and pregnane X receptor (PXR) are vital xenobiotic receptors activated by foreign substances, playing pivotal roles in regulating chemical metabolism. Traditionally, these receptors have been closely linked to their roles in mediating responses to toxic compounds. However, recent findings have revealed their newfound significance in maintaining gut homeostasis and regulating inflammatory processes.

**Aims:**

We investigated the dual roles of AhR and PXR activation. We started with dosage response experiments to determine optimal treatment conditions, such as treatment concentration and exposure time. Subsequently, we compared transcriptomic responses induced by chemical ligands with those from microbial metabolites, aiming to understand how AhR and PXR activation can yield both adverse and beneficial effects.

**Methods:**

Using mouse 3D colonoids and 2D monolayer cultures, PXR and AhR were activated using the microbial metabolites indole-3-propionic acid (IPA) and indole-3-pyruvic acid (IPyA), respectively. Concentrations were determined through dosage response experiments. Changes to PXR and AhR target gene expression were measured using qPCR. We compared the gene induction by IPA and IPyA to responses driven by the chemical ligands pregnenolone 16α-carbonitrile (PCN) and 2,3,7,8-tetrachlorodibenzo-p-dioxin (TCDD), respectively.

**Results:**

In 3D cell culture, PCN induced PXR target genes Cyp2c55 and Abcb1a, while IPA had no significant effect. Cyp3a11 was absent. Both IPyA and TCDD induced AhR genes, Cyp1a1 and Cyp1b1, with varying induction, and Cyp1a2 showed no response. In 2D cell culture, PCN significantly increased Cyp2c55 gene expression, but neither PCN nor IPA significantly induced Abcb1a. In 3D culture, TCDD significantly induced Cyp1a1 and Cyp1b1, while IPyA only induced Cyp1a1, revealing differences in AhR gene induction between 2D and 3D cell culture models.

**Conclusions:**

Our research reveals contrasting gene induction patterns following AhR and PXR activation through microbial metabolites and chemical ligands. These varied responses highlight the multifaceted roles these receptors play within the gut environment. This study enhances our understanding of xenobiotic receptors' contributions to maintaining gut homeostasis and regulating inflammatory processes, shedding light on the interplay between ligands and receptors.

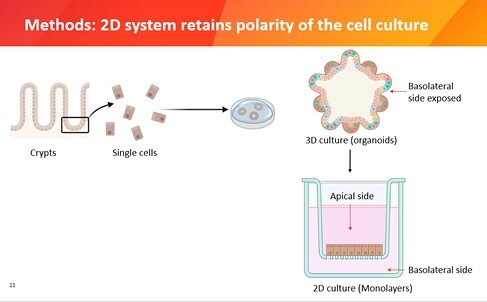

**Methods:**

First, we isolate the crypts of mice to create 3D organoids, were we have access to the basolateral side. To capture the true biological system, we convert the 3D culture to 2D. This enables us to explore both the apical and basolateral sides, retaining the crucial polarity of the cells.

**Funding Agencies:**

CIHR

